# Benign pulmonary epithelial inclusions within the pleura: a case report

**DOI:** 10.1186/1746-1596-2-21

**Published:** 2007-06-29

**Authors:** Barton Kenney, Marguerite Pinto, Robert Homer

**Affiliations:** 1 Department of Pathology, Yale University School of Medicine, 20 York St. EP-2, New Haven, CT 06510, USA

## Abstract

**Background:**

The normal visceral and parietal pleura are composed of a mesothelium-lined layer of fibrous connective tissue, consisting predominantly of collagen and elastin fibers, with interspersed nerves, lymphatics, and blood vessels. There is no normal epithelial component to the pleura, and the histologic finding of epithelial cells within pleural tissue typically indicates the presence of a malignant process. The one documented exception to this rule is the occasional occurrence of endometriotic implants, which can result in cyclic thoracic symptomatology and occasionally even hemothorax.

**Case Presentation:**

We present a case of seemingly benign epithelial inclusions within the pleura of a sixty year-old male patient who underwent exploratory thoracotomy and subsequent resection of bullous emphysematous blebs. Examination of the surgical specimen revealed well-defined nests of benign-appearing epithelial cells in glandular configurations at multiple sites within the pleura. These cells were immunopositive for TTF-1 and CK7 and immunonegative for calretenin, CK20, and CEA, indicating pulmonary epithelial derivation. Six months after resection, the patient is in stable health, with no clinical or radiologic evidence of bronchogenic carcinoma.

**Conclusion:**

To our knowledge, this is the first report of benign pulmonary epithelial inclusions within pleural tissue. It is important to be aware that benign pleural inclusions occur, so as to avoid confusion with more serious processes in a population of patients who may have oncologic risk factors.

## Background

Normal lung pleura is composed of fibrous connective tissue lined by mesothelium. While lymphatics, blood vessels, and nerves course within the pleural tissue, there is no normal epithelial constituent. Epithelial inclusions are occasionally seen in the form of endometriotic implants [1,2]. However, the presence of epithelial cells in the pleura is typically indicative of a malignant process, such as direct involvement by primary lung carcinoma, pleural metastasis, or lymphangitic spread of a carcinoma. We present a case of benign epithelial inclusions of pulmonary derivation within the pleura.

## Case presentation

We present the case of a 60 year-old male smoker with a history of chronic obstructive pulmonary disease (COPD) who presented with a two-week history of increasing shortness of breath and occasional wheezing. Review of systems was otherwise negative. Past medical history was remarkable for mild COPD, but the patient had otherwise been free of health problems. Physical examination of the chest revealed clear lung sounds bilaterally, and no other abnormal physical findings were evident. Chest x-ray demonstrated a right pneumothorax, and a subsequent CT scan confirmed a right pneumothorax (less than 10% of lung volume), a small right pleural effusion, and two large blebs in the right lung. No mass lesions were apparent. A nuclear medicine lung perfusion and ventilation scan showed retention of radiotracer consistent with COPD and heterogeneous patchy areas of perfusion, most likely related to air-trapping. It was decided to procede with surgical exploration and possible excision of the patient's emphysematous blebs. A right mini-thoracotomy was performed, and a large bleb was readily identified within the anterior chest, which was resected. A separate aarea of possible abscess was noted in the right apex, which on dissection by the surgeon contained yellow viscous fluid. Otherwise, no mass lesions or findings suspicious for carcinoma were encountered.

**Figure 1:**
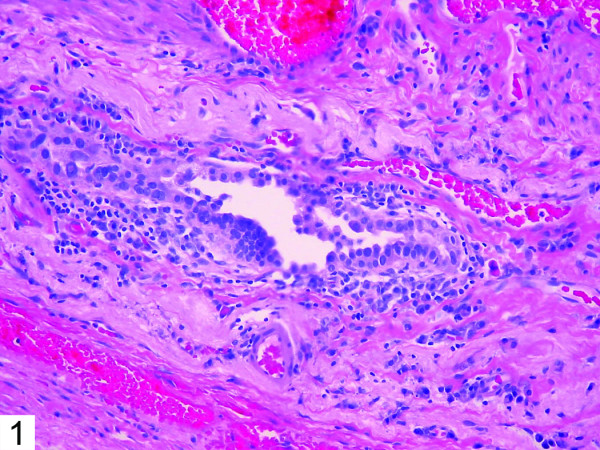
Epithelial inclusion within pleural tissue (H&E, 20×).

**Figure 2:**
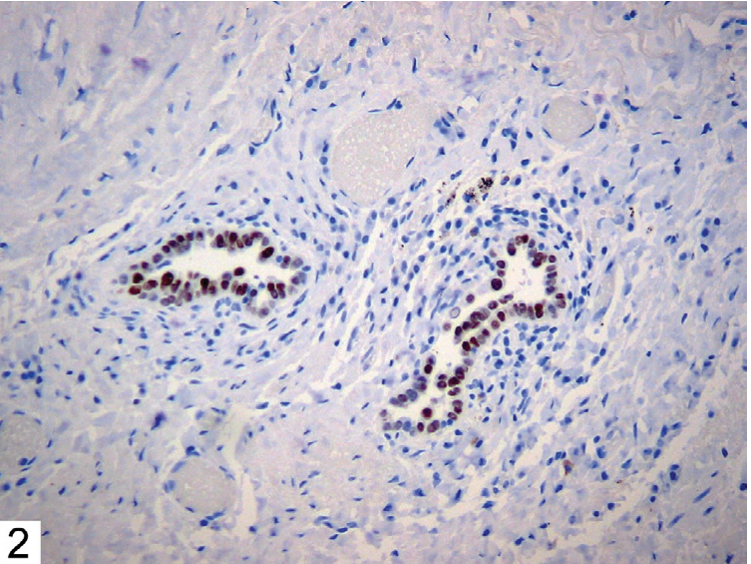
Immunohistochemical stain showing positivity for TTF-1.

**Figure 3:**
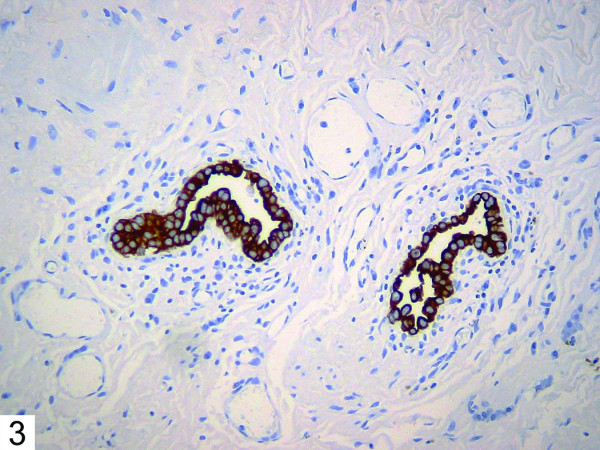
Immunohistochemical stain showing positivity for CK7.

**Figure 4:**
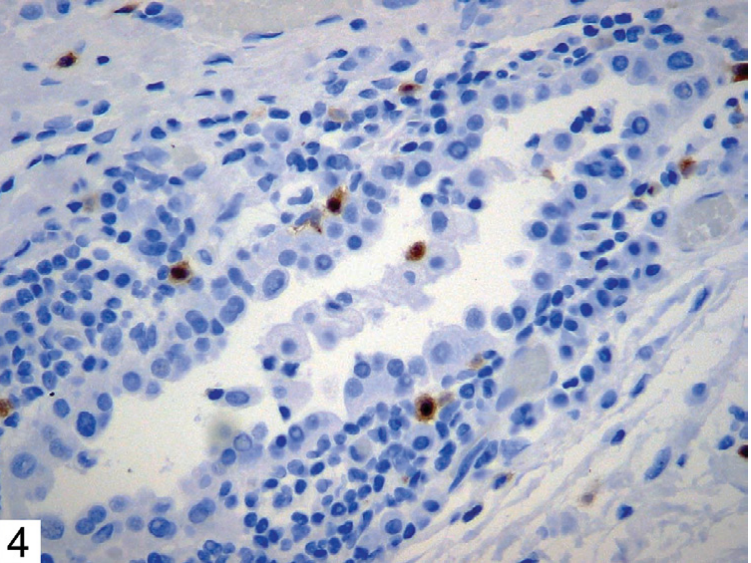
Immunohistochemical stain showing negativity for calretenin.

**Figure 5:**
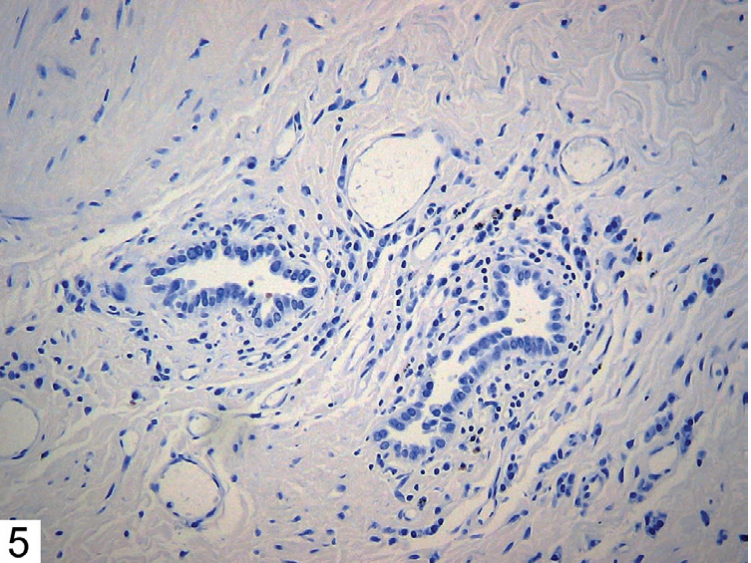
Immunohistochemical stain showing negativity for CK20.

Histologic examination of the patient's bullous resection revealed emphysematous changes and mild subpleural fibrosis. Additionally, multiple glandular structures were noted within the pleural tissue (Figure 1). These structures were composed of plump epithelioid cells with mild nuclear enlargement, fine chromatin, and inconspicuous nucleoli. No mitotic activity was evident. The glands were surrounded by fibrovascular tissue and showed no evidence of vascular or lymphatic invasion. The majority consisted of irregular small acinar structures, and there was no evidence of cribriforming or formation of solid structures. Immunohistochemical stains revealed positivity for TTF-1 (Figure 2) and CK7 (Figure 3), consistent with respiratory epithelial origin. Notably, calretenin (Figure 4) was negative, arguing against mesothelial derivation. Stains for CK20 (Figure 5) and CEA were also negative. The separately resected abscess showed fibrosis, subpleural bronchiectasis, and granulomata. There was no evidence of malignancy in any of the examined tissue.

Six months after resection, the patient is in stable health, with no clinical or radiologic evidence of bronchogenic carcinoma. His respiratory status remains compromised secondary to chronic lung disease. We feel that the identified epithelial inclusions represent a benign process, likely associated with the background of chronic obstructive lung disease.

## Conclusion

The pleural surfaces are subject to several pathologic processes which can result in the appearance of gland-like structures on histologic examination. Among malignant neoplasms, mesothelioma, bronchogenic carcinoma, and metastatic non-pulmonary carcinoma top the list [3-7]. Benign tumors also affect the pleura, including adenomatoid tumor and benign cystic mesothelioma [8-13]. However, relatively few benign processes have been shown to result in gland formation within the pleural tissue. The best documented example of such an entity is pleural endometriosis, which can result in endometrial gland and/or stromal deposition along or within the mesothelium [1,2].

Benign "ectopic" glandular inclusions have been reported most frequently in the lymph nodes, and these structures can be either epithelial or non-epithelial. As in the pleura, nodal endosalpingiosis/endometriosis is the most common example and has been identified in up to 41% of pelvic and para-aortic lymph node excision specimens in females [14-18]. Other reported inclusions within the retroperitoneal and intraperitoneal lymph nodes have included those of colonic and pancreatic origin [19,20]. Epithelial inclusions above the diaphragm are generally less common, but benign epithelial structures consisting of glandular breast tissue and squamous-lined cystic structures have been described in the axillary nodes [21,22]. A somewhat more controversial topic is the existence of benign thyroid inclusions in cervical nodes, although several compelling examples have been presented [23,24]. Non-epithelial inclusions are dominated by those composed of ectopic mesothelial cells which have been documented in the mediastinal and abdominal lymph nodes [14,15,25,26]. Finally, examples of benign nevo-melanocytic lymph node inclusions and decidual implants have been reported [27,28]. Examples of inclusions in non-nodal tissues are limited in number, but include peritoneal endosalpingiosis/endometriosis and surface epithelial inclusions of the ovary [17]. Some argue that peritoneal benign cystic mesothelioma is also an example of a benign inclusion process [11].

The histogenesis of epithelial inclusions is not well-understood. Within the lymph nodes, two theories are proposed. The first invokes entrapment of epithelial cells during embryologic development, i.e. embryologic maldevelopment [22]. The second is based on the concept of embolization, in which epithelial cells are exposed to and transported to lymph nodes via the lymphatics as a result of injury or inflammation [14]. Once in the lymph node, the cells are able to survive in the local milieu. Nodal endometriosis is the prototype of this model, as the inflammatory process of menstruation may allow epithelial cells access to the lymphatics. In mesothelium-lines tissues, dysembryogenesis is still a possible explanation. However, two additional processes are also considered. The first involves direct spread of epithelial cells to the mesothelium in the setting of effusion and inflammation. Similar to the case in lymph nodes, the non-native epithelial cells would then survive in their new environment. The other explanation invokes metaplastic proliferation of epithelial cells from the mesothelial surface derived from embryonal coelomic epithelium or from subcoelomic mesenchyme [15-17]. The potential stimulus for this metaplastic change is not known, but as in other circumstances, chronic inflammation may play a role. Regardless of the histogenic nature of inclusions, their clinical significance comes at the time of microscopic examination, wherein they may be mistaken for primary or metastatic epithelial malignancies [15,17,23,26].

The presented case represents the first report of benign pulmonary epithelial inclusions within the pleura. As in the examples above, the histogenesis of this process is not known. However, it may be surmised that chronic inflammation and/or fibrotic change in the setting of chronic lung disease may have contributed in some form, considering the patient's history of emphysema and subpleural fibrosis. The inflammatory millieu accompanying chronic lung disease and the process of repair, including secretion of growth factors such as PDGF, CTGF and TGF-Beta, may create a dysregulated environment suitable for abnormal stromal and epithelial proliferation. Alternatively, the glands may represent an unexplained metaplastic change or an example of embryologic dysregulation, leading to ectopic placement of epithelium. Simple entrapment of existing pulmonary tissue, however, seems unlikely, as the structures constitute well-formed glands rather than alveolar forms or other resident structures normally populating the most peripheral lung fields. Additionally, none of the several consulting pathologists who viewed this case were unable to convincingly explain their histologic appearance by simple entrapment. In fact, despite their relatively bland appearance, the presence of CK7 and TTF-1 positive glandular structures within the pleura raised concern for an occult lung carcinoma. However, there has been no clinical or radiologic evidence of malignancy in the patient thus far. Six months post-surgery he is in stable health and has exhibited no change in pulmonary symptomatology.

In summary, previous reports have described pleural epithelial inclusions consisting of endometriotic tissue, but there is no existing literature describing inclusions consisting of other benign epithelial tissues. In this case, benign pulmonary epithelial inclusions were incidentally identified histologically and confirmed by immunohistochemisical stains within the pleura of a sixty year-old male patient who underwent resection for bullous emphysema. The pathologist should be aware of the existence of such intrapleural structures, which may otherwise raise concern for a malignant process in patients with co-existing risk factors for bronchogenic carcinoma.

## Competing interests

The author(s) declare that they have no competing interests.

## Authors' contributions

BK investigated the topic and drafted the manuscript. RH conceived the report and contributed to manuscript drafting and editing. MP provided the diagnostic material and was involved in editing of the manuscript. All authors read and approved the final manuscript.
